# Extensive Sheep and Goat Production: The Role of Novel Technologies towards Sustainability and Animal Welfare

**DOI:** 10.3390/ani12070885

**Published:** 2022-03-31

**Authors:** Severiano R. Silva, Laura Sacarrão-Birrento, Mariana Almeida, David M. Ribeiro, Cristina Guedes, José Ramiro González Montaña, Alfredo F. Pereira, Konstantinos Zaralis, Ana Geraldo, Ouranios Tzamaloukas, Marta González Cabrera, Noemí Castro, Anastasio Argüello, Lorenzo E. Hernández-Castellano, Ángel J. Alonso-Diez, María J. Martín, Luis G. Cal-Pereyra, George Stilwell, André M. de Almeida

**Affiliations:** 1 Veterinary and Animal Research Centre (CECAV), Associate Laboratory of Animal and Veterinary Sciences (AL4AnimalS), University of Trás-os-Montes e Alto Douro, Quinta de Prados, 5000-801 Vila Real, Portugal; ssilva@utad.pt (S.R.S.); cguedes@utad.pt (C.G.); 2 LEAF Linking Landscape, Environment, Agriculture and Food, Instituto Superior de Agronomia, University of Lisbon, 1349-017 Lisboa, Portugal; laurasvbirrento@hotmail.com (L.S.-B.); davidribeiro@isa.ulisboa.pt (D.M.R.); aalmeida@isa.ulisboa.pt (A.M.d.A.); 3 Medicine, Surgery and Anatomy Veterinary Department, Veterinary Faculty, University of León, 24071 León, Spain; jrgonm@unileon.es (J.R.G.M.); ajalod@unileon.es (Á.J.A.-D.); 4 Mediterranean Institute for Agriculture, Environment and Development, Universidade de Évora, Apartado 94, 7006-554 Évora, Portugal; apereira@uevora.pt (A.F.P.); ageraldo@uevora.pt (A.G.); 5 Department of Agriculture, School of Agricultural Sciences, University of Western Macedonia, 53100 Florina, Greece; kzaralis@uowm.gr; 6 Department of Agricultural Sciences Biotechnology and Food Science, Cyprus University of Technology, Limassol 3036, Cyprus; ouranios.tzamaloukas@cut.ac.cy; 7 Animal Production and Biotechnology Group, Institute of Animal Health and Food Safety, Universidad de Las Palmas de Gran Canaria, 35413 Arucas, Spain; marta.gonzalez133@alu.ulpgc.es (M.G.C.); noemi.castro@ulpgc.es (N.C.); tacho@ulpgc.es (A.A.); lorenzo.hernandez@ulpgc.es (L.E.H.-C.); 8 Department of Animal Science, University of Lleida, 25198 Lleida, Spain; mariajose.martin@udl.cat; 9 Pathology Department, Veterinary Faculty, University of La República, Montevideo 11200, Uruguay; luiscalper@gmail.com; 10 CIISA—Animal Behaviour and Welfare Laboratory, Associate Laboratory for Animal and Veterinary Sciences (AL4AnimalS), Faculty of Veterinary Medicine, University of Lisbon, 1300-477 Lisbon, Portugal; stilwell@fmv.ulisboa.pt

**Keywords:** welfare, extensive, technology, PLF, sheep, goat, omics

## Abstract

**Simple Summary:**

New technologies have been recognized as valuable in controlling, monitoring, and managing farm animal activities. It makes it possible to deepen the knowledge of animal behavior and improve animal welfare and health, which has positive implications for the sustainability of animal production. In recent years, successful technological developments have been applied in intensive farming systems; however, due to challenging conditions that extensive pasture-based systems show, technology has been more limited. Nevertheless, awareness of the available technological solutions for extensive conditions can increase the implementation of their adoption among farmers and researchers. In this context, this review addresses the role of different technologies applied to sheep and goat production in extensive systems. Examples related to precision livestock farming, omics, thermal stress, colostrum intake, passive immunity, and newborn survival are presented; biomarkers of metabolic diseases and parasite resistance breeding are discussed.

**Abstract:**

Sheep and goat extensive production systems are very important in the context of global food security and the use of rangelands that have no alternative agricultural use. In such systems, there are enormous challenges to address. These include, for instance, classical production issues, such as nutrition or reproduction, as well as carbon-efficient systems within the climate-change context. An adequate response to these issues is determinant to economic and environmental sustainability. The answers to such problems need to combine efficiently not only the classical production aspects, but also the increasingly important health, welfare, and environmental aspects in an integrated fashion. The purpose of the study was to review the application of technological developments, in addition to remote-sensing in tandem with other state-of-the-art techniques that could be used within the framework of extensive production systems of sheep and goats and their impact on nutrition, production, and ultimately, the welfare of these species. In addition to precision livestock farming (PLF), these include other relevant technologies, namely omics and other areas of relevance in small-ruminant extensive production: heat stress, colostrum intake, passive immunity, newborn survival, biomarkers of metabolic disease diagnosis, and parasite resistance breeding. This work shows the substantial, dynamic nature of the scientific community to contribute to solutions that make extensive production systems of sheep and goats more sustainable, efficient, and aligned with current concerns with the environment and welfare.

## 1. Introduction

Sheep and goat extensive production systems are conducted in many different parts of the world, and they often use essentially marginal areas unsuitable for crop production, characterized by low productivity per animal and per surface area. They positively impact local socio-economic activities, playing an essential role in the maintenance of rural communities, on ecosystems, and in the production of unique, valued foods such as lamb meat or cheeses [1,2,3]. However, such systems are under significant pressure, mainly because there is little labor availability, and they have traditionally low productivity and often poor economic viability [2,4]. On the other hand, related to environmental and animal welfare, there is increasing pressure in many countries, especially in the European Union, on animal production in general, and on sheep and goat production in particular.

In extensive systems, such issues have been frequently overlooked due to the perception that they are more natural than intensive systems [5,6]. Nevertheless, sheep and goats in extensive systems face challenges that influence homeostasis and thus impact both production and welfare concurrently. Problems associated, for instance, with poor quality pastures that have strong implications for animal nutrition are one example. For example, undernutrition has significant impacts on pregnant ewes or goats [5,7,8], care at lambing, and neonatal mortality [9,10] or disease control [11]. Furthermore, on such systems, due to their frequently remote locations, animal monitoring is much more challenging to implement than in intensive production systems with confined animals [12]. This leads to a lack of human–animal contact that will make management difficult [6,13], causing it putatively to be a stressful event for the animal. Finally, harsh climate conditions on the range, such as high or low temperatures, droughts, snow or frost, high amounts of direct and indirect solar radiation, and humidity, also cause severe stress to the animals [14,15,16].

Recent reports point to farm management strategies that include, for instance, precision livestock farming (PLF) [17,18] among numerous other more classical approaches to dealing with such challenges. Although technological problems differ between extensive and intensive systems, such approaches are more common and applicable; the PLF approach, in particular, represents an opportunity to deepen the management in extensive systems and achieve the ambitious goal of real-time animal monitoring [19]. Indeed, real-time animal monitoring through PLF provides insight on animal welfare, particularly in situations dependent on a human intervention that call for action. These include, for instance, diseases and parasitism, imminent lambing, heat stress, or straying away from preferred areas and into areas notorious for predation [19,20]. In addition, there are also expectable gains in animal welfare surveillance and assessment protocols (e.g., AWIN) as well as legal obligations [21]. Finally, the ability to monitor the behavior of animals in extensive grazing areas could be used to improve pasture management [20,22]. 

Recently, and in the context mentioned above, Caja and co-workers have published a very interesting review [17] on the use of sensing solutions to improve the performance, health, and well-being of small ruminants. There are, nonetheless, other interesting approaches in addition to PLF that are of interest within the framework of small-ruminant, extensive production systems. Despite recent developments of PLF in sheep and goat production, there is a gap in the current state of knowledge on applying technology in the extensive systems of these species. In this sense, this review addresses, in addition to PLF, the roles of very different technologies which have been applied to various fields of animal science but not to sheep and goat extensive systems. In a broader view, these technologies are related to omics, thermal stress, colostrum intake and newborn survival, animal health, metabolic diseases, and parasite resistance. In this sense, the present review is divided into several sections. The first section addresses the issue of assessing animal welfare in small-ruminant-production extensive systems. The second section addresses examples of how different technologies have been used by the animal science community to address the most relevant issues. The first example deals with the application of PLF. The second example deals with omics in the context of small-ruminant nutrition and tolerance to seasonal weight loss. The latter is one of the most pressing issues in the framework of small-ruminant production in extensive production systems, particularly in the tropics and the Mediterranean. The third example addresses the problem of heat stress in small ruminants, also a significant issue in the context of production and welfare in extensive production systems. The fourth example addresses colostrum intake on a newborn’s survival. The fifth example is related to a production disease, pregnancy toxemia, and classical and novel approaches to monitoring it. Finally, the sixth section is related to novel technologies and breeding for internal parasite-resistance. All those examples use technologies that will positively impact production, health, and animal welfare, and make extensive systems more sustainable. Overall, the objective of the study was to review the application of technological developments that can be used within the scope of extensive systems and their implication in the production, health, and welfare of sheep and goats.

## 2. Sheep and Goat Extensive Production Systems: The Importance of Assessing Welfare

Extensive farming is generally perceived by society and consumers as a more sustainable and animal-friendly method of animal production. However, this remains to be validated by science. First, the welfare of animals kept, permanently or temporarily, in these systems must be scientifically and transparently studied [6,23]. Multiple approaches have been used to assess sheep and goat welfare in intensive systems [24], but ways for adapting and integrating this knowledge into protocols for small ruminants in extensive systems is still open to discussion. 

The first welfare assessment protocols using animal-based indicators were developed for intensive production systems, especially for dairy/beef cattle, poultry, and pigs (e.g., Welfare Quality), due to the overall high animal density per unit area associated with such systems. On the other hand, welfare assessment protocols for small ruminants, developed under the Animal Welfare Indicators (AWIN) project [25,26], were tested in different rearing conditions: dairy goats kept in intensive systems and sheep in extensive and semi-intensive farms [25]. Although these protocols may be useful for the assessment of welfare in all systems, it is necessary to ensure that appropriate and evidence-based changes are inserted and integrated. For example, some original measures may not be applicable or may show a very low prevalence and should thus be withdrawn, while new indicators will undoubtedly need to be added. Additionally, the assessors may need to record variable indicators dependable on various factors (e.g., terrain, distance, access, time, and weather constraints). Recently, a study [27] tested the feasibility and reliability of the AWIN protocol for the welfare assessment of dairy goats in semi-extensive farming conditions. It showed that some indicators from the AWIN sheep protocol could be successfully integrated into the original goat protocol, but some validation studies were nonetheless still needed.

Here, we discuss some of the main limits of these protocols when applied to fully extensive systems, as an opportunity for the use of novel technologies that are presented in the following sections.

Neither small-ruminant AWIN protocol includes males in the evaluation. Although this is not an issue for dairy cattle or pigs in intensive farms, it may be an important flaw for extensive and pasture farming systems [28,29,30]. Most published studies only assess the welfare and behavior of males in relation to the use of reproductive technologies [31] or in castration [32]. When applying an assessment protocol, it should be remembered that behavior is inherently different between females and males and that the presence of males may influence the behavior of the females (and vice-versa); for example, ewes are usually more active and vocal than rams [33,34]. Consequently, the welfare of males in the flock has often been overlooked. Therefore, developing or adapting protocols to groups that include males should consider such particularities. 

Some proposals have been made to adapt existing protocols to less intensive farming systems [13,27,35,36,37] although this should be regarded as a complex and challenging task considering the settings’ diversity [24,36]. Small ruminants bred in extensive farming systems are more exposed to weather conditions, which implies a need for considering temperature, humidity, and even wind exposure in welfare assessments. Extreme conditions, as well as sudden variations in weather, may significantly change the expression or intensity of some indicators and, thus, the welfare of the animals. Heat stress must be considered in these evaluations as well as each breed’s capacity to adapt and cope with its environment [15]. Breeds selected to live in extensive or pasture farming systems are usually more resilient and well adapted to the climatic conditions [38,39]. However, when exposed to temperatures outside their thermal comfort zone, more sturdy animals may mask poor welfare signs, making assessment very difficult [39]. The volatility of these circumstances poses an enormous and complex challenge for welfare assessment in small ruminant species kept in harsher environmental conditions. Nevertheless, indicators related to thermal comfort should be seen as an essential component of protocols to be used in extensive farming systems. For example, weather and environment monitoring stations may be important assets as they will allow for accurate correlations between welfare indicators and weather conditions. Other ways novel technologies may help in overcoming some of these issues will be further addressed in subsequent sections. 

One of the principles suggested by the AWIN protocol is good housing. Although in extensive systems actual housing is very limited or even absent, the provision of shade or shelter from wind, rain, or snow should be considered as the lack may result in very poor welfare, particularly for young animals. Providing some sort of shelter, whether for feeding or for protection from climate extremes, will result in higher welfare levels [40,41]. However, the extension and type of landscape in which these animals are generally kept may preclude the building of such shelters [24]. Thus, including the presence of shade/shelter and its accessibility, should be considered as an important indicator of animal comfort. 

Extensively kept small ruminants may be more prone to lameness problems due to constant exposure to wet soils and infection agents or due to the need to walk long distances along rough paths. Additionally, they are usually less frequently checked for signs of pain or discomfort and very rarely treated early, compared to animals in intensive farms [13]. Although conducting a thorough hoof examination poses great challenges in extensive systems, lameness assessments should include traditional gait scoring but also the careful examination of hooves if the prevalence of severely lame animals is detected. Moreover, the time of the year, climate, and terrain conditions should also be considered in these protocols. Technologies such as thermography, image scanning, or weight pads may be an expeditious and reliable way of detecting lame animals going through a race.

Several other problems deserve special attention in extensively kept flocks due to potential differences in prevalence and severity when compared with intensive systems [42]. Body condition score, diarrhea, and fleece condition are just a few examples of measures that may need substantiation and critical analysis before being approved [36,43]. These may vary according to weather conditions, season, and type of feeding (e.g., fresh grass), and the welfare impact of these changes must be verified. In extensive systems, in which the handling of individuals may be difficult, sorting gates and electronic weighing scales combined with e-ID may be used for the automatic collection of weight data. 

Well-designed studies on the behavior of small ruminants in extensive conditions are required to ensure the validity of the many animal-based measures, including the group mental state assessment trough the Quality Behavior Assessment (QBA) used in the existing AWIN protocols [44]. Despite the merit of on-farm welfare assessment schemes like the AWIN protocols, there is a need to introduce indicators that address positive aspects of animal welfare [45]. In this sense, other methods, including the five-domain model, were developed [46] and applied to sheep [13,47]. This model incorporates three survival-related domains (nutrition, environment, and health), a behavior-related domain, and a fifth domain that results from a comprehensive assessment of how the other domains impact the animal’s affective experience [48,49]. Besides, it is an ever-evolving model [50], which also allows for new interpretations and adaptations to extensively farmed species. In this way, identifying valid, reliable, and viable animal-based indicators related to the positive aspects of animal welfare will improve the quality of life of animals and strengthen communication about animal welfare to stakeholders [50]. Likewise, measuring human–animal interactions in extensive systems might differ from the intensive systems norm. Therefore, it is relevant to combine the knowledge acquired in intensive systems, but also to understand how the human–animal interaction is in extensive systems, and to not infer by mistake that the applicable behavioral parameters might be the same.

An equally important issue in applying protocols to extensive systems is feasibility. For example, the time needed to apply the protocol in very large settings, or the difficulties associated with the exposure to open-field constraints, all have to be considered to ensure validity and feasibility. In this context, on-farm welfare assessment feasibility can be increased by adopting a strategy supported on a few valid and reliable animal-based indicators [13] complemented by the introduction of new technologies, such as automatic-recording devices or drones [51]. 

Finally, a word on an often-demoted issue—the need for specifically trained evaluators [27,52] so that the repeatability and credibility of the protocols are guaranteed. Experienced and competent auditors in intensive system assessments may not be prepared for the work needed in extensive settings. 

In summary, welfare assessment of extensively kept small ruminants should be seen as a very specific subject, and not just an extension or simple adaptation of the protocols validated for intensive systems. Due to particular features and limitations, full-field assessment may be too difficult to manage through traditional farm-level personal observation. This provides excellent opportunities for new sensor technologies, as will be discussed in the following sections. The main constraints to the use of new technologies in small ruminants in extensive settings, such as drones, intra-ruminal sensors or ear-tags containing accelerometers, are cost and the difficulty of getting accurate, real-time readings. 

## 3. Animal Welfare in Small Ruminant under Extensive Production Systems: The Role of Novel Technologies

In this section, we conduct a comprehensive review of the technologies that are being applied to sheep and goats in extensive systems and their impact on welfare. Examples will be given of precision livestock farming (PLF), the application of omics technologies, technologies applied to thermal stress, the role of colostrum intake on newborn survival, animal health, metabolic diseases, and breeding for parasite resistance.

### 3.1. The Use of Precision Livestock Farming Applied to Sheep and Goat Extensive Production

Technological developments that have been applied to sheep and goat extensive production systems, although very diverse, can be framed within the concept of PLF. PLF has been described as the use of real-time monitoring technologies to manage the smallest manageable production unit [53]. PLF uses equipment, data, and software that allow individual animal information to guide decisions and inputs more precisely in an animal production system [17,18]. As mentioned, PLF approaches critically depend on identifying the animals individually, and electronic identification (EID) allows the achievement of this goal. EID has undergone significant developments since the early 1980s and is typically linked to the use of tags or boluses [17]. In 2004, the European Union made EID mandatory for all sheep and goats [21], and it currently represents an opportunity to further increase the scope of PLF technologies into extensive management systems [18]. EID is linked to technologies such as global positioning systems; behavior–activity loggers; virtual fencing; stationary management systems, such as walk-over-weighing systems; and automatic drafters [17,18,54]. These technologies allow for the precise management of sheep and goats, individually, in small groups, and as a flock [53,54]. Individual animal performance provides support for better decision making, which could benefit animal performance, economic performance, labor [21], and animal health and welfare [55]. As these management systems develop, vast amounts of data can be collected from thousands of farms, further assisting and directing agricultural policies on sheep and goat production, global warming mitigation, and antibiotic resistance [18]. Furthermore, such precision data can be used and integrated to find solutions to disease, welfare, productivity, and environmental issues and improve farming outputs [55,56,57]. Also, positive economic results have been observed in different reports [21,58]. As extensive systems are very diverse, there are many circumstances in which PLF is not suitable or even feasible. In any case, despite promising results, most of the technologies have not yet reached an applicability level similar to those introduced in intensive systems [17]. It should also be considered that cultural dynamics, financial stability aspects, confidence in new technologies, and the openness of farmers to new ideas do not always encourage wider adoption of innovative technologies in sheep and goat extensive systems [18,59]. In this sense, and considering that PLF is a collection of relatively novel technologies, the effects on animal welfare in extensive systems are not yet apparent [56]. However, it is expected that PLF solutions will play a key role in assessing welfare in extensive systems and will be driven by a greater capacity of technologies to recognize welfare and, more significantly, whether the welfare of farm animals is improved by the application of technologies [56]. Nevertheless, available solutions that include PLF approaches have been used to assess various issues related to sheep and goats’ health, behavior, and welfare in extensive systems. For a brief overview, please refer to Table 1, which summarizes the PLF research to assess behavior, lambing, live weight, and lameness in sheep.

### 3.2. The Use of Omics Applied to Sheep and Goat Extensive Production

Omics refers to the use of novel molecular biological approaches that allow for the profiling of a particular organism, tissue, or cell concerning its genes (genomics), mRNA transcripts (transcriptomics), proteins (proteomics), and metabolites (metabolomics) at a particular point in time [73]. Post-genomic platforms, namely proteomics, metabolomics, and transcriptomics, are gaining importance in the context of animal production, and more recently, the integration of these different platforms with food and nutrition science have been demonstrated to be a very interesting asset to obtain an in-depth analysis on animal physiology, production, and other related fields of animal science [74]. Despite many studies concerning animal welfare in the behavior and ethology fields, the establishment of biomarkers can be a great complement to improving the welfare assessment [75] and the knowledge of animals’ physiological processes and regulatory mechanisms of adaptation to harsh conditions [76]. Overall, we can consider that the different omics are a valuable tool for addressing several key aspects of small-ruminant science, particularly in the framework of production and welfare in extensive sheep and goat farming. However, there is an aspect that is particularly associated with small-ruminant-production systems. It is related to the year-round fluctuations in the rain pattern that in turn cause important changes in the availability and quality of pasture and fodder for ruminants, particularly those in the extensive systems. Indeed, the occurrence of a dry season that can last several months leads to the unavailability and lignification (decreased nutritional value) of pasture during such months. In turn, this leads to seasonal weight loss (SWL), a problem to which several small ruminant breeds have adapted over the selection process. This issue is particularly pertinent in the framework of this review and will therefore be described as a case study in this section. SWL is one of the most pressing issues in extensive animal production in tropical and Mediterranean regions. There are two solutions to address this problem. First, supplementation with additional feed is often problematic, if not impossible, to implement in the large areas that characterize these extensive systems. Second, the most cost-effective approach is using breeds adapted to feed scarcity [77]. To select such breeds, novel technologies are available, allowing for the identification of biomarkers and molecular patterns related to SWL resilience. Several studies using omics have been conducted over recent years [78]. Here, we will focus on two examples where omics were used to study SWL: meat-producing sheep in Australia and dairy-goat production in the Canary Islands. 

Sheep production in Australia is mainly based on extensive systems, primarily designed for wool production using the Australian Merino (AM) breed. In recent years, these production systems have been increasingly exposed to droughts that compromise animal welfare and the economic viability of farms due to undernutrition [79]. Moreover, AM sheep are highly susceptible to myiasis (caused by blowfly strikes), which compromises their health and welfare [80]. To deal with this issue, farmers routinely remove the hind-quarters skin folds which are susceptible to blowfly strikes in a surgical procedure called *mulesing* [81]. In addition to decreasing wool prices and the consequently reduced profitability of AM flocks, these welfare concerns have motivated a shift in these production systems. Indeed, producers have been steering towards meat production, particularly destined for live animal exports bound for the Middle East and Asia. Because the AM sheep is primarily bred for wool production and is also highly susceptible to SWL and external parasites, it is less appealing for meat production compared to South-African breeds such as the Dorper. The latter is a composite breed conceptualized for meat production, originating in the breeding of Persian Blackhead and Dorset Horn. In addition to this breed, using fat-tailed breeds (e.g., Damara, another South-African breed) poses another alternative, taking advantage of their superior fat depots to endure SWL [82]. To evaluate the response of the AM, Dorper, and Damara to SWL, a live-animal trial was carried out to induce weight loss experimentally [83]. Since then, several different analytical approaches have been carried out to assess the physiological response of these breeds. Briefly, the restricted groups of Damara and Dorper lost a smaller percentage of their initial live weight (LW) than the AM group. Unrestricted animals increased by 7%, 13%, and 10% of their initial LW, respectively [83]. The differences between breeds extended to carcass and meat characteristics, with both South-African breeds having heavier carcasses, higher fat deposition, and darker meat compared to the AM breed [84]. The different muscle development inherent to each breed was reflected in the muscle proteome [85,86]. The muscle structure of the Dorper breed is particularly affected when restricted, lowering the abundance of contractile apparatus proteins, such as myosin and tubulin. In addition, a higher number of cellular functions were impacted in the AM breed as a consequence of SWL, such as ATP and actin binding [85]. This was corroborated by a metabolomics [87] and amino acid [88] profiling analysis of the muscle tissue, which identified lower levels of amino acids (e.g., tyrosine, glycine, and taurine) in the muscle of the AM breed, suggesting lower muscle growth and increased endogenous protein mobilization compared to the other two breeds. Interestingly, the Damara breed was seen to increase the abundance of structural proteins such as desmin because of SWL [86]. This highlights an increased resilience of Damara sheep under SWL, where they counter-balance muscle amino acid mobilization [88] by attempting to maintain structural integrity. The liver proteome of these sheep has been studied, including the mitochondrial proteome [89,90]. These studies revealed that the Damara breed under SWL mobilizes more lipids than the AM breed through the higher abundance of lipid transport proteins, such as apolipoprotein E, and lipid metabolism proteins, such as annexin. In turn, the unrestricted group has a metabolism oriented for the synthesis of fatty acids, particularly branch-chain fatty acids, which accumulate in the tail [91]. Indeed, the mobilization of tail fat under SWL is the distinct mechanism of the resilience of the Damara breed against SWL. Its mobilization has caused the increase of fat tissue mineral concentrations [92] since the presence of fat has a diluting effect on the tissue mineral profiles [93]. The quality of wool from the AM breed was also negatively influenced by SWL, which caused a reduction in fiber diameter and an increase of the high-sulfur protein KAP13-1 and the glycine–tyrosine-rich KAP6 family of proteins [94]. The data mentioned above demonstrate the physiological mechanisms behind the improved adaptation of SWL of the Dorper and Damara breeds compared to the AM breed. Moreover, several different biomarkers have emerged from these studies that can be used to choose hardy breeds whose welfare is not so negatively affected by current conditions.

Dairy-goat production in the Canary Islands is another example where the reared animals are subjected to SWL, particularly in the easternmost islands, which are very dry compared to the western islands with a more temperate climate. The different rainfall in La Palma (a humid island) and Fuerteventura (a dry island) has an impact on the available pasture, and consequently, on animal production. The Majorera goat from the latter island has been adapted to weight loss, whereas the Palmera goat from the former is more susceptible to feed restriction, which threatens welfare in dairy production systems. Similar to the sheep example described above, a trial was conducted to compare the response of both of these breeds to SWL. Restricted groups lost 13% of their initial live weight and 87% of their initial milk yield [95]. This had repercussions on the FA composition of milk and the mammary gland, particularly for the Palmera breed, where restriction increased oleic acid and reduced palmitic acid in the secretory tissue, whereas the Majorera had no differences [96]. Despite this, feed restriction caused the mobilization of endogenous FA in both breeds, as indicated by higher levels of circulating non-esterified FA [97]. However, omics approaches have revealed that the response was different in the two breeds, with more resistance features in the Majorera. Indeed, a transcriptomics approach identified a wide set of genes with differential expression in the mammary gland caused by SWL. The restricted Majorera increased the expression of genes related to amino acid, lipid, carbohydrate, and nucleoside transport, indicating reduced metabolic activity. Contrarily, the restricted Palmera goats upregulated genes involved in suppressing cell differentiation and related to the response to DNA damage, demonstrating the effects of mammary gland involution. Comparing both restricted groups identified two genes associated with unregulated tissue development in Palmera goats (CPM and ASB11) [98]. This is confirmed through two different proteomic approaches that identified a high abundance of apoptotic proteins in the restricted Palmeras and suggested cadherin-13, collagen alpha-1, and clusterin as another set of putative biomarkers to SWL tolerance in the Majorera goat breed [99,100]. The detrimental effect of feed restriction extended to the metabolome of the mammary gland and milk [101]. Restricted groups had lower AMP, ADP, ATP, and IMP, all energy-related molecules characteristic of low metabolic rates in both restricted breeds. In addition, feed restriction influenced the rumen metabolism, which seems to have contributed to the lower levels of Krebs-cycle intermediates (citrate, fumarate, succinate) in the milk of the restricted goats. So, as SWL can represent a problem in the dairy sector, it is essential to establish biomarkers to ensure the health-status monitoring, apply new breeding systems, and essentially guarantee animal welfare [76,102]. Similar to the Australian sheep studies, these goat studies have yielded several putative biomarkers of SWL-resistance that could be used to select animals for enhanced response to SWL. This is particularly important given that the climate is rapidly changing, and susceptible breeds could soon be subjected to harsh droughts that threaten not only animal welfare related to undernutrition, but also local food security and economies.

**Figure 1:**
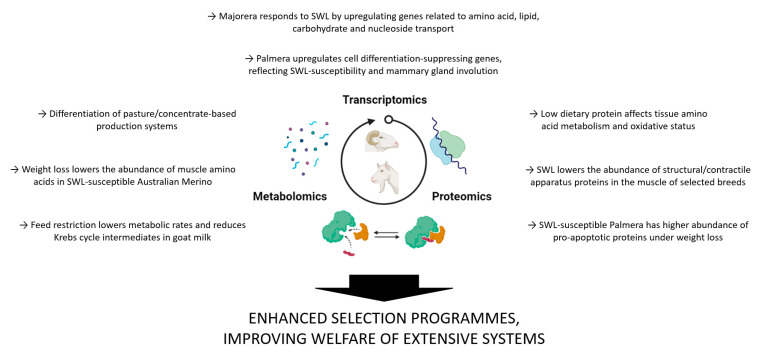
Omics information was obtained within the context of small-ruminant production systems, comparing dairy goat breeds (Majorera and Palmera) and meat-producing sheep (Australian Merino, Damara, and Dorper) under seasonal weight loss (SWL). Image created using biorender.com.

The information obtained from these omics approaches provides a detailed look at the impact of SWL on a molecular level (Figure 1). This allows for a deeper understanding of the metabolic response to weight loss differentiation among adapted and susceptible breeds in two distinguished contexts: dairy and meat production. The identified differences are supported by classical approaches, including mineral, amino acid, and fatty acid profiling. Identifying biomarkers for SWL-resistance enables the improvement of breeding programs for the selection of hardy breeds towards the economic viability and welfare of animals in the extensive production systems in tropical and Mediterranean regions.

### 3.3. Novel Technologies and Thermal Stress in Sheep and Goat Extensive Production

Climate change poses severe threats to livestock production, particularly in extensive small-ruminant production systems. A negative impact on livestock production and productivity are expected with the temperature increase and the frequency of extreme weather. Consequences will likely be even more severe in arid and semi-arid grazing systems where higher temperatures and lower rainfall are expected [103]. 

In this changing climate scenario, environmental challenges stimulate behavioral responses in animals. The welfare status is directly linked to livestock behavior, and hence change in the behavioral pattern will help determine urgent environmental conditions. As the behavioral response is the first step animals take to cope with the heat load, studying animal behavior is valuable to understanding how to best use and design strategies to cope with the environment. However, the impact of heat stress and the animals’ responses are difficult to predict and analyze; thus, a better understanding of the response of the animals under heat stress is required. Several technologies and methodologies have thus been proposed to monitor small-ruminant temperatures under field conditions, as subsequently detailed.

Automated monitoring of behavior using digital technologies might increase labor efficiency. It has been suggested that such technologies would allow farmers to better monitor and manage animals, resulting in a higher efficiency in production, lower environmental impact, and improved animal welfare [104]. Technological advancement has made this task more accessible with the help of recording devices. Other tracking devices. Such as access-control systems, lasers, video systems, and video systems combined with GPS and cell phones have also been attempted in livestock research [105,106]. GPS tracking devices offer the potential to monitor behavioral measures, such as shelter-seeking, remotely and to determine how shelter availability influences paddock utilization by ewes [107]. These products can benefit the farmers as they can provide some rudimentary surveillance of the sheep. For example, during hot summer weather, night grazing increases as animals seek shade during hot hours of the day. Another recent technology development has been integrating radio frequency identification (RFID) sensors capable of monitoring animal behavior [108]. RFID sensors can provide permanent individual identification of animals, and readers can identify several animals simultaneously. In addition, the RFID technology allows the frequency and duration of animal visits to the feed bunk and water troughs to be recorded, and this can be used to assess feeding and water intake behavior. While these products can be of significant help, they have some limitations. Indeed, the sampling frequency is relatively low, and wooded areas also make it challenging to locate the exact position of the animals [109]. Even so, the information provided only by the GPS is insufficient; knowing where the animals are in the pasture does not provide information about their behavior or activities. However, the simple behavioral classification used in conjunction with GPS tracking in sheep has led to the distinguishability between “active” and “inactive” behavior [22]. GPS used in combination with accelerometers allows for the identification of more complex behaviors, such as rumination, movement, grazing, standing, walking, lying down, and running [110,111]. A recent study mentions that raw accelerometry can be used to predict discrete numerical signatures associated with sheep’s grazing, resting, and walking activities. This type of sensor can be a very effective tool for identifying sheep grazing activity [112].

The rectal temperature has long been used to evaluate core temperature and quantify the heat stress response in livestock. An extensive review on automatic data collection in heat-stressed sheep refers that manual clinical thermometry is not appropriate for assessing circadian patterns of body temperature in free-range animals or those in extensive grazing systems [113]. The vagina is another good site to collect temperature. Vaginal temperature (VT) sensors correlate with rectal temperature measurements [114]. Notwithstanding, attention must be paid to the changes in vaginal blood flows during the estrous cycle and gestation stages because the vaginal temperature can change accordingly [115]. Devices such as rectal probes have the advantage over traditional thermometry as they enable producers to measure temperature remotely. Rectal probes record core temperature most consistently, particularly in male animals, despite a relative degree of invasiveness. Therefore, it is likely that the stability of rectal probes could limit the accuracy of temperature data in sheep [116,117]. A range of subcutaneous microchips and other implantable devices is also being developed to measure body temperature in livestock continuously. Typically, microchip transponders are injected under the skin and activated through a handheld receiver, where the temperature reading is then relayed instantaneously [113]. The intra-peritoneal, or intra-abdominal, area is the most common site for implants. The temperature loggers surgically implanted into the peritoneum display with accuracy the subtle body temperature changes in sheep exposed to hot conditions [113].

Intra-ruminal temperature sensors have arisen as a non-invasive alternative to the surgical implantation of devices. Temperature loggers consisted of a chip, antenna, and battery built into an orally administered bolus. This technology enables real-time data collection through instant wireless transmission [118] or stores the information until the animal is in the vicinity of a receiving antenna [119]. Rumen temperature is highly correlated with core temperature, even though the ruminal temperature is higher than the core temperature by approximately 2 °C. This difference can be reduced (by almost 1 °C) during fasting and drinking episodes [120,121]. 

The literature is scarce despite the potential impact of rumen temperature sensors to investigate heat stress in sheep or goats [122]. Infrared thermography, which measures real-time surface temperature distribution, is a tool that is being increasingly used in farm animals due to society’s growing interest in animal welfare. From a physiological perspective, the differences in the thermal image reflect changes in blood flow that allow thermal exchange between an animal’s skin and the environment through vasoconstriction or vasodilatation [123]. However, studies conducted in this field often lead us to question the usefulness and viability of the thermal windows currently suggested for ruminants since certain anatomical aspects, such as hair, skin color or the lack of it, and skin thickness, can affect specific thermal windows, making them unviable in these species [124,125]. 

Concerning well-insulated wool sheep, it is known that environmental heat exchange is profoundly affected by fleece length. One effect of shearing is that it causes the skin to thicken, which influences heat transfer at the skin’s surface [126]. In a high-temperature environment, it is essential to understand better the adaptive capabilities of animals and use the appropriate tools to quantify their responses to heat stress. On the other hand, to improve welfare, it is necessary to mitigate the effects of high radiant temperatures and high solar radiation that can trigger severe heat stress. In extensive grazing systems, controlling the ambient temperature is impractical. Providing some shade for the animals and sufficient water is usually all that can be achieved. Shade trees reduce heat stress on animals and help increase productivity [127]. Several studies have shown the beneficial effects of providing shade or shelter to sheep to decrease the heat gain by solar radiation. Studies have found that shaded sheep are more productive and have less severe physiological responses than unshaded sheep [128]. The magnitude of these effects depends upon the height, the canopy type, and the trees’ density [129]. In general, silvopastoral systems are considered beneficial for animal welfare, despite the variability of tree arrangements [130]. Silvopastoral systems are attractive alternatives for reducing heat loads and increasing animal thermal comfort. Sheep reared in silvopastoral systems tend to increase their time in grazing and reduce by 10% their water consumption [131]. The projected shade of trees with denser foliage offers better protection against solar radiation. However, beyond the dimension and position aspects of shade, it is crucial to consider the adequate protection given by any shade.

In the Mediterranean, the protection against solar radiation of pine trees (Pinus genus), olive tree (Olea europea) and cork and helm oaks (Quercus ilex and Quercus suber) is very distinct, with better shade quality usually associated with cork oak trees. Silanikove [128] found that, in the summer Mediterranean region, unshaded sheep had a respiration rate that was 56% higher than that of sheltered sheep due to the effect of direct solar radiation. However, Johnson [132] found that unshaded and shaded animals all maintained similar body temperatures and respiratory rates, despite the possible advantages of shading. It was thus suggested that wool length greater than 20 mm might have substantially slowed radiative gain. Providing shade, either natural or artificial, in regions or production systems prone to heat stress problems seems thus to be a practice to be implemented and encouraged.

Improving the welfare of domesticated ruminants subjected to climatic change is a new challenge. Therefore, future selection in small ruminants should aim to balance heat adaptation, health, and production. The main objective of future selection to sustain small-ruminant production should focus on several specific adaptive characteristics [133], preferably combining heat tolerance with SWL tolerance as detailed in the previous section. Essential traits to consider in such selection could be skin and hair type, sweat gland capacity, reproductive rate, feed conversion efficiency and drought tolerance, and metabolic heat production. In addition, the adapted local breeds could be a gene bank alternative as an appropriate bio-resource to sustain small-ruminant production under changing climatic conditions.

### 3.4. Novel Technologies and Colostrum Intake on Lamb and Kid Survival

Most mammals are born with an immature immune system [134]. Therefore, they depend on the transfer of maternal immune components to obtain the necessary protection against external agents, such as pathogenic bacteria or viruses. This process is known as passive immune transfer (PIT). In some mammals (i.e., humans, rabbits, and mice), PIT is mainly achieved via the placenta [135]. However, the synepitheliochorial or epitheliochorial placentation in ungulate species does not transfer immune components (mainly immunoglobulins) through the placenta [136]. Therefore, newborn ruminants strictly depend on colostrum intake a few hours after birth to be immunized until the immune system of these animals is ready to synthesize the immune components [137]. Colostrum is the most important source of nutrition for newborn mammals, and it contains a complex mixture of components such as fat, proteins, lactose, and minerals [138]. Besides the nutritional function, colostrum also contains a wide range of proteins that actively protect the neonate against pathogens and other postpartum environmental challenges [139]. Its composition can be highly variable and influenced by nutrition and hormones [140]. Immunoglobulins are a major constituent of colostrum. In ruminants, the major immunoglobulins present in colostrum are IgG and IgM. However, colostrum composition can affect the degree of immunity acquired by the newborn. Indeed, several studies have shown how IgG and IgM plasma concentrations rapidly increase in newborn kids and lambs during the first 24 h postpartum after being fed with high-quality colostrum (i.e., IgG > 50 g/L) within the first 2 h after birth [141,142]. Other colostrum components, such as the complement system proteins or the chitotriosidase enzyme, have also been suggested as contributing to the early protection of the newborn [141]. Colostrum intake is therefore particularly important in sheep and goat extensive production systems. As these animals are in most cases raised on the range, lambs and kids are very dependent on the dams for colostrum and milk intake. This is particularly noticeable when compared, for instance, with ruminants raised under intensive systems. In those systems, farmers can better control colostrum and milk intake by the newborn animals. As such, research on extensive sheep and goat production systems that aims to ensure and monitor adequate colostrum intake by newborn animals is required. 

Inadequate colostrum feeding management (i.e., low colostrum quality, reduced volume, and delayed first intake after birth) can have negative short-term effects on mortality and the use of antibiotics [141,143]. However, it can also have long-term consequences as animals receiving an inappropriate colostrum feeding management will be at higher risk for suffering infections. Consequently, these animals have lesser development, and therefore, lower performance (i.e., reduced milk yield) when they reach adulthood. In extensive production systems, the control and management of the animals is more difficult when compared to intensive production systems [5]. This fact also affects birth monitoring and appropriate care of the newborn animal. In these systems, mortality rates in kids and lambs are between 7.5% and 15% and 13% and 25%, respectively [6,144]. Multiple factors can increase mortality rates in intensive and extensive production systems. Some of these include low birth weight, short gestation periods, large litter sizes, poor mothering, and hypothermia, as well as other unexpected events around birth [140]. In addition, extensive systems are highly variable and heterogeneous in terms of climate conditions and food and water availability and quality [145]. Thus, these factors can raise serious welfare concerns related to chronic hunger, thirst, and thermal stress [6]. These adverse conditions directly affect the quality of colostrum and milk produced by the dams. In addition, the control of high-quality colostrum ingestion is a challenge for this type of production systems. 

The most accurate techniques used to measure colostrum quality, based on IgG concentration, are the radial immunodiffusion assay (RID) and the enzyme-linked immunoassay (ELISA) [146]. However, both are laboratory techniques which are not available at the farm level. On-farm techniques, such as densimeters specifically designed to measure colostrum density (i.e., colostrometers), visual assessment, or refractometers are suitable tools for estimating colostrum IgG concentration [147]. Several studies have validated the use of the BRIX refractometer to measure the percentage of total solids and indirectly estimate IgG concentration in colostrum [148]. However, the use of these tools in extensive systems is difficult to implement as it requires a degree of animal handling that is not feasible under extensive conditions. Therefore, one way to assess health status and assess colostrum and milk production abilities in a flock is to control animal body condition score. This aspect can be combined with the use of automated weighing systems that provide higher frequency of BW measurements, as well as the use of pedometers which monitor animal activity during grazing. Low body condition score compromises the immune function and increases the risk of health problems during lactation [149]. Another way of monitoring the animals on the range could be, for instance, the use of remotely controlled aerial vehicles, usually called drones [20,150]. PLF advances have been made for identifying and monitoring parturition-related behaviors of sheep. For this, information on the location and movement obtained with GNSS tracking collars [65,151] or accelerometers for ewe changes of activity [66,152] were used. The promising results found in those works will help increase vigilance for parturition time and early detection of dystocia, improving animal welfare, and decreasing lamb mortality [153]. Finally, observing the dams and the offspring allows the detection of abnormal behavior such as lack of licking by dams of newborns or reduced protective behavior from the dam to the newborn, which may indicate an unsuccessful bonding and therefore a failure of the passive immune transfer. These techniques, although relatively simple, can provide insights into the behavior of the animals and monitor colostrum intake and the lactation in concentrated lambing or kidding seasons with minimal interference. They are thus extremely interesting in answering such issues and will undoubtedly need to be considered in this context.

### 3.5. Novel Technologies and the Diagnostics of Metabolic Diseases in Sheep and Goat Extensive Production: Pregnancy Toxemia

Pregnancy toxemia (PT) is a metabolic disorder that affects pregnant sheep and goats during the last trimester of pregnancy, especially in the last weeks, due to the body’s inability to maintain energy homeostasis and the negative energy balance that occurs in these females [154,155]. Energy requirements in late pregnancy, especially when there is a multiple gestation, or even with a large fetus, increase above maintenance levels by approximately 150% in ewes with a single pregnancy and up to 200% in ewes pregnant with twins [156]. The most widely accepted theory for explaining the pathogenesis of this disease proposes a failure in the mechanisms regulating energy homeostasis. In fact, and added to the significant increase in energy requirements, there is a significant decrease in food intake, especially dry matter, mainly as a cause of the reduction in ruminal capacity due to the increasing volume of the pregnant uterus [154,156]. Consequently, and to compensate for the energy deficit, pregnant sheep and goats are forced to mobilize their fat reserves, causing an increase in the levels of ketone bodies. This disease is essentially a severe form of ketosis, characterized by low circulating glucose and high levels of ketone bodies in the blood [154,155,156,157,158].

The disease occurs more frequently in lean (BCS < 2 on the 5-point scale) or obese (BCS ≥ 4) females, as well as in animals with two or more fetuses [159]. This disease has been associated with feeding in pastures in extensive systems when adverse weather conditions (cold, snow, heavy rain, etc.) make it difficult for sheep to feed. Other factors include an advanced age of the female, poor dentition, foot processes, gastrointestinal and liver parasites, or any other liver disease [154,156,158]. Although the incidence and economic consequences of this disease are not easy to quantify [157], there is some information on its impact specifically in sheep and goat production [160]. In addition to mortality, there is a significant impact on the health and production of affected sheep, with either clinical or subclinical disease. The number of affected sheep varies from a few individuals to 40% of the flock [154], and it can lead to the death of 80% of affected sheep [156]. It also causes dystocia and complicated births, with retention of fetal membranes, metritis [161,162,163], and even mastitis [162], as well as lambs with less vitality that often die within a few days [158,164].

Both sheep and goats with gestational toxemia show digestive and neurological signs. In the initial phase, they present signs that often go unnoticed. They are apathetic and slow, falling behind and separating themselves from the rest of the herd. As the depression increases, they remain immobile; they do not react to the presence of humans or dogs, suffer the loss of auditory and ocular reflexes, have difficulty walking, move in circles, and tend to press their heads against objects [155,156,157,164,165]. In the more advanced stages, they show myoclonic contractions of the head, chest, and extremities [155], with convulsive episodes [156], evolving towards sternal or lateral decubitus, comatose states, and death in the majority of untreated animals [155,156].

Traditionally, a significant decrease in glucose, cholesterol, total protein, albumin, globulin, T3, T4, calcium, sodium, and potassium, or insulin has been established in females with pregnancy toxemia [158]. In addition, significant increases in triglycerides, non-esterified fatty acids (NEFA), β-hydroxybutyrate (BOHB), cortisol, AST, ALT, GGT, LDH, urea, and creatinine have been found [158] (Table 2). Most clinical signs of PT are a consequence of hypoglycemic encephalopathy [157]. Hypoglycemia, which also affects fetal glycemia [160], results in fewer lambs born alive and lower fetal weights at birth, and hinders the viability of the lamb, and therefore, increases perinatal mortality [158,162,166]. Given that early identification of a metabolic imbalance could lead to improved treatment success, Cal-Pereyra et al. [167] propose the following values for blood glucose (1.59 ± 0.24 mmol/L; 28.62 ± 4.33 mg/dL), BOHB (2.26 ± 1.03 mmol/L), and plasma cortisol (15.09 ± 7.75 nmol/ L, 5.47± 2.81 ng/mL) to diagnose a case of ovine PT early, even when the sheep do not show clinical signs.

Other, less conventional parameters have been measured in animals affected by gestational toxemia, and among them we can point to fructosamine or glycosylated hemoglobin; serum concentrations of potassium, sodium, and calcium; and the presence of metabolic acidosis in the blood or aciduria (measured in urine) (Table 3).

Currently, other markers are being used in those sheep that present symptoms of pregnancy toxemia, such as the so-called acute phase proteins (APPs), among them, fibrinogen, serum amyloid A (SAA), haptoglobin (Hp), and glycoprotein α-1 acid. The significant increase in APPs found in sheep with gestational toxemia could be related to the changes in lipid metabolism that occur in this process [168]. SAA concentrations were not significantly altered in goats with subclinical toxemia but did increase significantly when goats had clinical pregnancy toxemia [169]. Serum haptoglobin increased significantly in toxemic sheep and goats [168,169]. Previous studies have reported that there is a significant correlation between Hp and BHB in subclinical pregnancy toxemia in goats [169]. C-reactive protein (CRP) was significantly reduced in energy-restricted sheep, presumably due to decreased hepatic synthesis, although a large increase in C-reactive protein levels has been reported in toxemic pregnant sheep [178].

Although the exact pathogenic mechanism of pregnancy toxemia is not known, Yarim et al. [179] propose a key role for cytokines and chemokines, including interleukin-1β (IL-1β), tumor necrosis factor alpha (TNF-α), and monocyte chemotactic protein-1 (MCP-1), proving an increase, related to the severity of the disease, so they could be used to monitor the prognosis of pregnancy toxemia in sheep [179]. Albay et al. [169] observed a strong increase in TNF-α activity in the serum of toxemic goats, and especially in clinical toxemia.

Likewise, biomarkers of myocardial injury, such as troponin I (cTnI) and the cardiac isoenzyme creatine kinase (CK-MB), were measured in sheep and goats affected by PT, with a finding of significantly higher serum concentrations in sick females [180,181]. All this indicates that females with PT show a consecutive cardiac injury, so that the increase in cardiac biomarkers could be considered good diagnostic and prognostic indicators in sheep and goats affected by pregnancy toxemia [180,181].

Until a few years ago, the assessment of ketone bodies in blood (and also in milk) was complicated to perform, but currently we can determine the presence of ketonuria (ketone bodies in urine) semi-quantitatively using test strips [165], or in blood using handheld meters, which are very easy to use, very sensitive, and reliable, which facilitates the diagnosis of TPO at the field level in a very economical way [182,183]. Ketones are detected in the urine when blood levels already exceed 0.7 mmol/L [156].

In conclusion, the toxemia of gestation that affects some sheep and goats in advanced stages of pregnancy causes important changes in the conventional blood profile. Currently, other, less conventional parameters have been evaluated, but with great importance in the early diagnosis and prognosis of pregnancy toxemia, and both in clinical and subclinical disease. These last parameters cannot always be implemented in some countries or in certain production systems. However, they should be considered mainly in small, dairy ruminants.

### 3.6. Novel Technologies towards Parasite Resistance in Small Ruminant Extensive Production

Gastrointestinal (GIN) parasites are one of the major health problems in small ruminants, particularly in pasture-based production systems. A common manifestation of this disease is a temporary depression in voluntary feed intake, i.e., anorexia, which impairs the production efficiency and welfare of the periparturient ewe (late pregnancy and early lactation) and parasite-naïve, growing lambs [184]. Anthelmintic drenching is the usual control method, but the multi-drug anthelmintic resistance of gastrointestinal nematodes observed in small ruminants all over the world raises concerns regarding the future of drug efficiency [180,181,182,183,184,185]. Furthermore, new classes of anthelmintic substances are unlikely to provide a permanent solution to this problem since resistance to new drugs can develop quickly [181,182,183,184,185,186], let alone public concerns about pharmaceutical use and their residues on products and the environment [187,188].

Research efforts and practical applications resort to combined anthelmintic drug usage methods and alternative anthelmintic control measures. These alternative methods can be divided into those using nutrition, genetics, or other interventions resulting from inhibiting the contact between susceptible animals and infective parasitic larvae. Moreover, towards this direction, systems that deploy integrated management and monitoring of pasture performance and grazing livestock activities, similar to the approach that has been proposed for climate-smart agriculture [189] could also be of importance in controlling nematode parasitism in grazing ruminants, particularity in extensive or pasture fed/organic systems. For example, it has been proposed that altering the grazing behavior of infected animals or other activity patterns, such as lying time, could potentially be utilized for early detection and monitoring of nematode parasitism infections [190,191]. Such advances could be integrated into holistic management systems that fall within the concept of precision livestock farming [192].

A well-studied alternative method for reducing GIN parasitism in small ruminants is the use of bioactive forages, i.e., forages rich in secondary metabolites, reviewed by [193]. Such secondary compounds are only found in some plant species and belong to a wide range of substances, such as tannins, lactones, alkaloids, saponins, terpenes, glycosides, and phenolic compounds. Although these compounds may have been reported to cause negative effects on intake, metabolism, or trigger nutritional deficiencies, when fed in moderate quantities, they have shown potential for controlling GIN in small ruminants [194]. Their mode of action has been proposed to be direct, on the cuticle of the parasite as an anthelmintic, or indirect, through enhanced host response, affecting the animal’s resistance to parasites and, therefore, reducing their population or enhancing animal resilience to parasitism (limiting production losses while it persists). The direct mode of action is attributed to the presence of secondary plant compounds in gut digesta damaging the cuticle of the adult parasite, as has been detected using electron microscopy [195], and therefore affecting the viability and/or fecundity of parasites within the animal or larval development and survival in the feces [196]. The indirect mode of action is proposed particularly for tanniferous plants since the condensed tannins are able to bind to proteins in the rumen, limiting microbial digestion, and then disassociate in the abomasum and duodenum, providing by-pass protein supply and enhancing host response. Furthermore, an alternative method of indirect action involves a likely immunostimulant attribute of the secondary metabolites, either locally or systematically [197], but this mode of action needs further investigation. Regardless of the manner of action, many tanniferous plants have been tested and have shown a potential anti-parasitic effect in sheep and goats in several studies over the last 20 years. Therefore, a targeted list of the most established plant species could include *Lespedeza cuneata* (Sericea lespedeza), *Hedysarum coronarium* (sulla), *Onobrychis viciifolia* (sainfoin), *Lotus pedunculatus* (big trefoil), and *Lotus corniculatus* (birdsfoot trefoil), as reviewed by [193]. However, due to the variability observed in the plant secondary compounds depending on the weather, the season, and/or the geographical area, bioactive plants’ effects on animal parasitism are very variable. Therefore, this method could not stand alone but should be combined with other control measures such as proper grazing management, targeted anthelmintic drenching, or feed supplementation [196].

It was shown that the use of copper oxide wire particles (COWP), initially administered for copper deficiency, also affects GIN parasitism [198], and thereafter numerous studies have demonstrated its effectivity against different gastrointestinal species and particularly against *Haemoncus contortus* [199]. The mode of action is more likely direct due to the increase of copper concentration in the abomasum digesta and potential damage on the cuticle of parasites residing in this organ although an indirect action, through the increase of the host’s copper status, has also been proposed [200]. Nevertheless, the use of this alternative method shows greater effectivity when combined with other methods, such as anthelmintic use [201] or tannin-rich forages [202]. Even though the copper particles can be found in the abomasum of small ruminants for many weeks after administration, its anthelmintic effectivity may not persist for more than 41 days after administration [203]. Nevertheless, using this alternative method may result in increased copper concentrations in the livers of sheep and goats [204], and therefore low doses of 0.5 or 1 g per day are recommended and only under veterinary supervision [205].

It has long been recognized that nutrition can influence the outcome of exposure of sheep to nematode parasites. Most research on the effects of nutrition on immunity to gastrointestinal nematodes in sheep has been concentrated on metabolizable protein (MP). This happens because many components of the immune effector responses are highly proteinaceous in nature [184]. In addition, damaged epithelial cells resulting from the infection must be replaced, imposing an increased need for protein synthesis [184]. Consequently, there is an increased protein demand for the host, especially because of the reduced feed intake as a result of the infection [184,206]. During the early acquisition phase of immunity against nematode infection, dietary protein supplementation enhances the ability to acquire and express immunity in growing lambs [207]. In the case of adult sheep, numerous studies have demonstrated that supplementation with protein in late pregnancy or early lactation, or both, can reduce the fecal nematode egg output and/or worm burdens in ewes, while protein scarcity exaggerates the extent of the consequences of infection [206,208]. Moreover, the periparturient relaxation of immunity observed in ruminants is influenced by nutritional demand, being greater in animals carrying or rearing twin lambs compared with singles, and the response will be moderated by the extent of body protein reserves [208]. In addition, protein supplementation can also affect the degree of anorexia in nematode-infected ewes during the periparturient period in a similar way to its effects on the periparturient relaxation of immunity [206].

Breeding management is also an effective way of controlling parasitic nematode infections in small ruminants [209]. The notion behind this approach lies in the fact that host resistance to nematode infections is an inherited trait, with a heritability index that varies from 0.1 up to 0.4 [210]. Animals that show an inherited resistance to nematode infections normally have fewer adult nematodes, more inhibited larvae, and shorter and less fecund adults. In practice, this is reflected in the low number of parasite eggs per gram of feces (fecal egg counts, FEC), despite being infected with a sufficient number of infective larvae [210]. Therefore, animals displaying an intrinsic resistance to parasites can improve this trait in the next generations but are also less likely to contaminate the grazing pasture with high levels of parasitic eggs, protecting the less resilient or resistant animals within the flock [209]. Accordingly, differences in resistance to nematode infections have also been observed between breeds or different genotypes of sheep. Breeds that have been selected for increased growth rates or fleece weights have been found to have higher FEC than randomly bred animals [210,211], while differences in the extent of the periparturient relaxation of immunity in ewes also exist between different breeds or genotypes [206]. It has been proposed that this variation is very likely to be associated with variation in the production potential [206,211] although it appears that there is no general relationship between resistance to parasites and productivity within a breed [209]. Comparison of immune function between breeds that have been selected for genetic resistance with unselected breeds suggests that the genetic variation in response to infection is likely the result of the mechanisms that regulate the prioritization of nutrient use between immune defense and production traits [206]. This is not surprising given the evidence that genetically resistant sheep have more significant numbers of mast cells and globule leucocytes in the abomasal and intestinal mucosa and a greater production of parasite-specific immunoglobulins during infection with intestinal nematodes [212].

Additional methods have also been proposed. Nematophagous fungi, for instance, have the potential, as a biological measure, to reduce the free-living larval stages of GIN in livestock feces and to consequently reduce the infective larvae ingested by grazing animals [213,214]. Most of the research to date has concentrated on the fungus Duddingotonia flagrans, which is able to survive passage through the gastrointestinal tract and, under suitable conditions, provide high larvae-trapping efficacy, as shown in early studies in sheep [213,214] and goats [215]. The mode of action occurs through the formation of a variety of trapping structures that destroy developing parasitic larvae in feces [216]. This form of control is environmentally friendly as this fungus does not affect native, free-living nematodes in the soil and is not detectable in the environment 2 months after treatment [217]. The main delivery method is through mixing feeding supplements with fungal spores during daily feeding. Animals have thus the ability to consume an adequate amount of feed/spore mixture, usually beginning at the start of the grazing season, as these applications have shown effectivity in numerous studies, as reviewed by [218], and have recently been approved as commercial products within the EU [219].

Lastly, grazing management is a long-known alternative method that can influence GIN infection in sheep and goats. Rotational grazing strategies for sheep, allocating them to new plots and returning them to previously grazed paddocks after an adequate time needed for the lessening of the infective larvae’s presence, could reduce the incidence of parasitism compared to continuously grazed plots [216]. However, the efficiency of this method depends on the precise information available on the local epidemiological conditions regarding the life cycle of the dominant parasitic species [220]. The concept of reduced infective larvae consumption by small ruminants through grazing management includes the use of browsing for goats, the use of plant species with complex leaf structures that do not accommodate larval movement, the use of multi-species forages, and/or the alternate grazing of cattle or horses with sheep and goats [221]. However, grazing methods are usually applied combined with deworming applications, and this has to be guided to avoid the speedy development of anthelmintic resistance within the parasitic population, in other words, to use the available drugs wisely so that a small portion of susceptible parasites remain within the parasitic population [222].

## 4. General Conclusions and Future Prospects

The sustainability of extensive systems for sheep and goats will depend on improving their efficiency and productivity for meat, milk, and fiber production. Like all livestock production, extensive systems face enormous challenges that include environmental responsibility and sustainability, the ways that plant and animal resources are used, and how animal welfare is answered.

The extensive systems of sheep and goats are very diverse. They are often associated with poor-quality grazing, harsh weather conditions, very rudimentary fences or other infrastructures, and the limited use of equipment to foster animal management. Under such conditions, different constraints and limitations are likely to occur. They include animal welfare, nutritional scarcity, heat stress, neonate survival, metabolic diseases, and parasitism. Such factors significantly aggravate the skilled labor constraints, limit work efficiency, and do not allow for the frequent assessments of animals.

Over the last few decades, the scientific community and field technicians have used different technologies to address such huge challenges. These include, for instance, PLF and animal welfare monitoring; the use of omics as a selection tool for seasonal weight loss tolerance; remote temperature monitoring; regular flock body condition scoring; blood parameter measurements in pregnancy toxemia diagnosis; and directed supplementation with specific compounds for GIN tolerance, in addition, of course, to specific feeding and selection strategies.

This review shows that introducing novel technologies into sheep and goats extensive systems renders it possible to monitor the welfare of animals due to greater precision in detecting and anticipating problems associated with health, lambing, nutrition, and management. There are already robust results in applying many such technologies in other species (e.g., pigs, poultry, and dairy cattle). The results presented in this review show a way to find solutions for extensive sheep and goat farming systems. Although addressed separately in the majority of the scientific and technical approaches, their implementation must nonetheless be seen as a whole and from a global perspective. That is perhaps the most daunting challenge. Nevertheless, it is mandatory for the future of sheep and goat production in extensive systems. The animal science research community can only address such challenges through scientific cooperation and high levels of integration with stakeholders, such as farmers, field technicians, veterinarians. and animal production specialists, and ultimately consumers and policy regulators.

## Figures and Tables

**Table 1:** Summary of research work to assess behavior, lambing time, lameness and live weight in sheep by kinematic and kinetic approaches.

WMT	n/Breed	Aim	Technique	Results	Ref.
B	Cheviot ewes	Determine if different behavior types associated with grazing	Sensor accelerometer-integrated collars	Accuracy 90%	[22]
B	29 Scottish Blackface ewes	General activity and circadian rhythm of activity with sheep body weight change	Sensor accelerometer-integrated collars		[60]
B	3 Merino	Behavioral and movement patterns of individuals	Tri-axial sensors, temperature sensor, and GPS	Accuracy > 75%	[61]
B	6 sheep	Continuous surveillance of eating behavior for monitoring ruminant health, productivity, and welfare	Tri-axial gyroscope and tri-axial accelerometer	Accuracy > 86%	[62]
B	50 Merino	Low-cost solution to monitoring of the location of all the animals in a herd and the continuous updating of location data	GPS collars (25 ewes) and BLE (25 ewes)		[63]
B	10 Norwegian White	System that automatically generated individual animal behavior and localization	Real-time sensor tags and tri-axial accelerometers (ST LIS2DE)	SE = 98.16% (standing); SE = 100% (lying)	[64]
B	Serra da Estrela breed	Autonomous system to control sheep posture and monitor their location in real-time.	Collar set of sensors (inertial and ultrasound) and a microcontroller and actuators (i.e., stimulation devices, namely sound and electrostatic)		[65]
La	40 ewes	Predictive model to identify the day of lambing in extensive sheep	GNSS tracking collars	Accuracy 83.0%, SE = 63.6%, SP = 84.1%	[66]
La	39 Merino	Monitor changes in sheep behavior around the time of lambing	Accelerometer ear-tags (Axivity AX3)		[67]
L	10 Merino Poll Dorset ewes	Ability of a tri-axial accelerometer to discriminate between sound and lame gait	Accelerometers (GCDC X16) on 3 points: neck collar, ear, and leg	Accuracy 82% (ear), 35% (collar), and 87% (leg)	[68]
L	20 various breeds	Relationship between sheep hoof-health status and the load a sheep distributes to each hoof	Hoof weigh crate raceway two strain-gauge cantilever load cells	SE = 100%, SP = 95%	[69]
LW	4 flocks	LW as an indicator of nutritional status	WoW	Repeatability 0.20–0.76	[70]
LW	900 ewes	Ewe performance of two different methods of feed allocation	Automatic weigh and drafting crates coupled with EID technology	Accuracy 52%	[71]
LW	Romane ewes	LW data were recorded as each ewe entered voluntarily and walked throughout the WoW	WoW	Accuracy 0.89 and 0.98	[72]

WMT: Welfare/management target; B: behavior; La: lambing; L: lameness: LW: live weight; GPS: global positioning system; SE: sensitivity = true positive/(true positive + false negative) × 100; SP: specificity = true negative/(true negative + false positive) × 100; BLE: low-cost Bluetooth low energy tags; WoW: walk-over weighing, GNSS: global navigation satellite system; EID: electronic identification; Ref: reference.

**Table 2:** Variables traditionally measured in the diagnosis of pregnancy toxemia in sheep and goats.

Parameter	Reference Interval and Problematic Values	References
Glycemia	50–70 mg/dL (2.8–3.9 mmol/L); falls below 20 mg/dL	[155,158,165,168,169]
Blood ketone bodies (especially ß-hydroxybutyrate (BOHB)	<1.1 mmol/L;	[154,155,156,158]
	>2 mmol/L (36.03 mg/dL);	[170]
	>5.0 mmol/L (90.09 mg/dL);	[154,158,159,164,165]
	19.0 mmol/l.	[171]
BOHB in aqueous humor or cerebrospinal fluid	>2.5 mmol/L (45.0 mg/dL) y > 0.5 mmol/L (9.0 mg/dL), respectively.	[172,173]
Total proteinemia, albuminemia and globulins in blood	Significant fall, due to liver injury and anorexia; possible false increase due to dehydration.	[164,168,174]
Urea, blood urea nitrogen (BUN) and serum creatinine	Increased urea, blood urea nitrogen (BUN), and serum creatinine, due to renal dysfunction.	[154,155,168,175]
Serum cortisol and thyroid hormones (T3 and T4)	Increased cortisol, justified by hyperactive adrenal glands.	[164,168]
	Decreased T3 and T4, due to hypersecretion of cortisol.	[168,176]
Blood enzymes: aspartate aminotransferase (AST); alanine aminotransferase (ALT); gamma glutamyl transferase (GGT); lactate dehydrogenase (LDH) and creatine kinase (CK)	Increased AST, ALT, GGT, LDH, and CK, possibly due to liver dysfunction. Positive correlation between AST activity and the degree of hepatic vacuolization, a consequence of hepatic steatosis, therefore this enzyme could be used as an early and reliable indicator of liver damage in sheep with clinical pregnancy toxemia [177]	AST: [155,164,171,174,175,177] ALT and GGT: [168,174,175] LDH: [168] CK: [164,175]

**Table 3:** Other, less conventional parameters measured in ewes and goats affected by pregnancy toxemia.

Parameter	Modification of the Values and Justification	References
Fructosamine and glycosylated hemoglobin	Both indicate not the current glycemia, but over a long period prior to measurement; low values suggest persistent hypoglycemia.	[154,164]
Minerals: potassium (K), sodium (Na), calcium (Ca) and phosphorus (P) in blood	Large decrease in K, Na and Ca.	[168]
	No changes in phosphorus.	[168]
	Due by starvation, dehydration, metabolic acidosis, electrolyte imbalance and renal dysfunction, as well as increased lipolysis that can induce hypocalcemia.	[156,168]
	High calcium demand of late gestation leads to a significant decrease in maternal calcemia.	[154]
Metabolic acidosis and aciduria	Metabolic acidosis (lactate and pyruvate measured in blood).	[164,175]
	Aciduria, measured in urine using semi-quantitative test strips.	[164,165]

## Data Availability

Not applicable.
